# LncRNA-SVUGP2 suppresses progression of hepatocellular carcinoma

**DOI:** 10.18632/oncotarget.18279

**Published:** 2017-05-29

**Authors:** Jiangfeng Hu, Chenlin Song, Bensong Duan, Xiaoyan Zhang, Dongliang Li, Liang Zhu, Hengjun Gao

**Affiliations:** ^1^ Department of Gastroenterology, Tongji Hospital, Tongji University School of Medicine, Shanghai, China; ^2^ Division of Molecular Biology of the Cell II, German Cancer Research Center, DKFZ-ZMBH Alliance, Heidelberg, Germany; ^3^ Shanghai Engineering Center for Molecular Medicine, National Engineering Center for Biochip at Shanghai, Shanghai, China; ^4^ Department of Hepatobiliary Medicine, Fuzhou General Hospital of Nanjing Command, PLA, Fuzhou, China; ^5^ Department of Gastroenterology, Changzheng Hospital, Second Military Medical University, Shanghai, China; ^6^ National Engineering Center for Biochip at Shanghai, Shanghai, China; ^7^ Department of Gastroenterology, Institute of Digestive Diseases, Tongji University School of Medicine, Wuhan, China

**Keywords:** lncRNA-SVUGP2, hepatocellular carcinoma, proliferation, invasion, liver

## Abstract

Numerous studies indicate that long noncoding RNAs (lncRNAs) are dysregulated in hepatocellular carcinoma (HCC) and might serve as potential diagnostic biomarkers and therapeutic targets of HCC. Therefore, it is interesting to globally identify the lncRNAs altered in HCC. In our study, we used microarray to profile the levels of lncRNAs and mRNAs in three pairs of HCC and their adjacent noncancerous samples. We found lncRNA-SVUGP2, which is a splice variant of the UGP2 gene, was down-regulated in HCC samples and correlates with a better prognosis in patients with HCC. Overexpression of lncRNA-SVUGP2 in HepG2 and Hep3B liver cancer cells suppresses cell proliferation *in vitro* and tumor growth *in vivo*. Moreover, lncRNA-SVUGP2 suppresses the invasion ability of liver cancer cell lines and downregulates the mRNA and protein levels of MMP2 and 9. Additionally, lncRNA-SVUGP2 positively or negatively correlates with many mRNAs in liver cancer tissues, indicating it is multifunctional in regulating carcinogenesis.

## INTRODUCTION

Hepatocellular carcinoma (HCC) is one of the most common types of liver cancer, accounting for 70 to 85% of liver cancer cases in most countries [1]. HCC is currently the third leading cause of cancer deaths worldwide, causing 600 thousand deaths per year [2, 3]. The highest death rates are found in eastern and southeastern Asia and in central and western Africa [4]. The pathogenesis of HCC is multifactorial, including infection with hepatitis B virus (HBV) or hepatitis C virus (HCV), alcohol consumption, hepatic toxins, non-alcoholic fatty liver disease, and genetic modification [5, 6]. Furthermore, cellular phenomena also contribute to HCC initiation and progression, such as tumor microenvironment, inflammation, oxidative stress, and hypoxia [7]. Additionally, numerous key signal transduction pathways have been identified as associated with the pathogenesis of HCC, including the Raf/MAPK/ERK pathway, PI3K/Akt/mTOR pathway, Jak/Stat pathway, and WNT-β-catenin pathway and so on [8–10].

Recently, a number of studies indicated that long noncoding RNAs (lncRNAs) are dysregulated in HCC. For example, lncRNA HULC is up-regulated in HCC and associated with its grade. Among the tumor tissues, higher HULC expression is positively correlated with HBV-positive status [11]. LncRNA HOTAIR and HOTTIP are also up-regulated in HCC. High levels of these lncRNAs indicate reduced overall survival and poorer prognosis [12–14]. More than ten lncRNAs have been reported as up-regulated in HCC to date [15, 16]. In addition to the three lncRNA mentioned above, other up-regulated lncRNAs include TUC338 [17], MALAT1 [18, 19], MDIG [20], UFC1 [9], and CCAT1 [21, 22]. In addition, some lncRNAs are down-regulated in HCC. For example, lncRNA MEG3 is down-regulated in HCC tissues and cell lines. Low MEG3 levels promote tumor growth and prevent cell apoptosis [23]. In addition, lncRNA-LET is also down-regulated in HCC. Low lncRNA-LET levels are correlated with increased HCC metastasis in HCC patients. Altogether, lncRNAs play important roles in HCC carcinogenesis and serve as potential prognostic markers in HCC diagnosis.

LncRNAs are a class of non-protein-coding transcripts that are longer than 200 nucleotides [24–26]. Accumulating evidence indicates that lncRNAs are tightly related to tumorigenesis [27]. Genome-wide association studies in different cancer types verify that greater than 80% of cancer-associated single nucleotide polymorphisms (SNPs) are present in noncoding regions of the genome [28]. In addition, lncRNAs participate in numerous biological processes involved in carcinogenesis, such as cell proliferation, cell migration, and tumor metastasis [29–32]. In addition, the multiple molecular mechanisms of lncRNAs in cancer are currently attracting increasing attention. LncRNAs regulate the level of oncogenes or tumor suppressor genes and further affect biological functions [23, 33]. The important and interesting roles of lncRNAs in carcinogenesis may be attributed to their multiple functions in gene regulation, targeting transcriptional activators or repressors and RNA polymerase II [34, 35]. LncRNAs induce chromatin remodeling and epigenetic modification via recruitment of chromatin modifying proteins, such as polycomb repressive complex 1 (PRC1) and 2 (PRC2) [36] and DNA methyltransferase DNMT1 [37]. Moreover, lncRNAs titrate miRNAs away from their targets and act as miRNA sponges [38]. Considering the critical role of lncRNA in cancer, the identification of important lncRNAs in cancer and developing lncRNA-based therapeutic strategies would be meaningful in the future.

In our study, we used microarray to globally profile lncRNAs and mRNAs levels in three HCC and their paired adjacent noncancerous samples. Interestingly, we found that lncRNA-SVUGP2 is decreased in HCC samples and correlates with better prognosis. In addition, overexpression of this lncRNA inhibits cell proliferation and invasion of the liver cancer cell lines HepG2 and Hep3B, suggesting lncRNA-SVUGP2 is a potential predictive marker and exhibits tumor-suppressive effects in HCC.

## RESULTS

### Global profiling of lncRNAs and coding genes in HCC cancer tissue and noncancerous tissue

**Figure 1:**
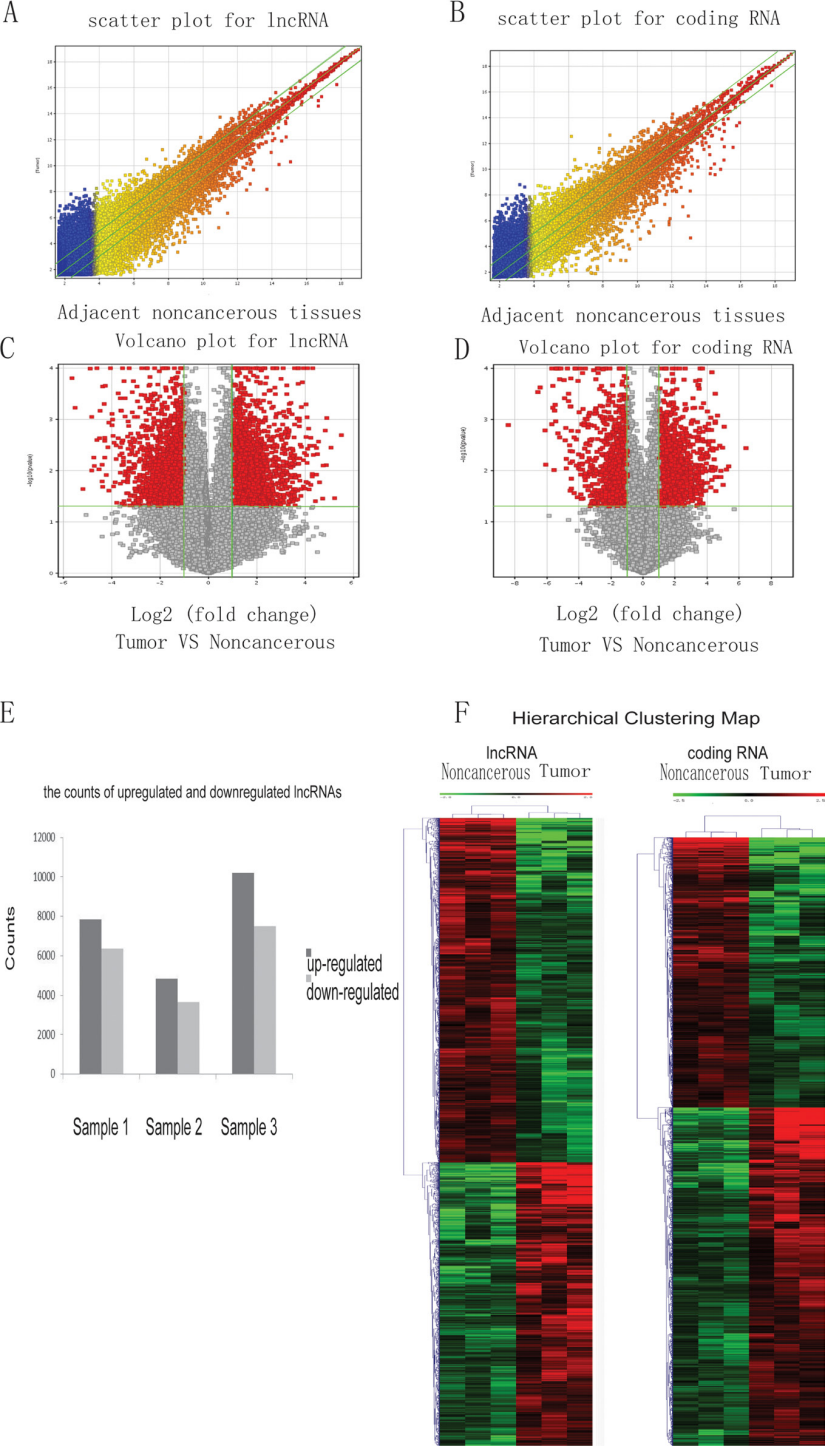
The expression profiles of lncRNA and coding genes in HCC and paired adjacent noncancerous samples Total RNA from 3 pairs of HCC and noncancerous samples was isolated to study gene expression using the human lncRNA microarray v4.0. A scatter plot was used to assess lncRNA (**A**) and coding gene RNA (**B**) expression reproducibility. The values on the x and y-axis of the scatter plot are the averaged normalized signal values of noncancerous and tumor tissue (log 2 scaled). Additionally, a volcano plot was used to analyze the differentially expressed lncRNAs (**C**) and coding genes (**D**) between noncancerous and cancer tissues. The red dots to the left and to the right of the vertical green lines indicate RNAs with a greater than 2.0-fold change and statistical significance (*p*-value < 0.05). (**E**) The counts of upregulated and downregulated lncRNAs in the three samples used in the microarray. (**F**) Hierarchical Clustering Map of abnormal lncRNA and coding RNA in HCC.

To identify the lncRNAs and coding genes dysregulated in HCC, RNAs extracted from three pairs of HCC and their adjacent noncancerous samples were analyzed using a custom microarray (OE Biotech Human lncRNA Microarray v4.0 4 × 180K). This microarray contains 78,243 lncRNAs and 32,776 coding genes. The reproducibility of these three pairs of microarrays was confirmed by scatter plots (Figure 1A, 1B). Volcano plots were used to identify the lncRNAs and mRNAs changed in HCC tissues (Figure 1C, 1D). A volcano plot is constructed by two values, fold-change values, and *p*-values, indicating variance and statistical significance respectively. Therefore, it is a good tool to select the lncRNAs and mRNAs significantly and reliably dysregulated in HCC tissues.

Given the important roles of lncRNAs in HCC, we sought to identify the most dysregulated lncRNAs in HCC. In these three pairs of samples, an average of 7620 lncRNAs (range 4824 to 10195) was up-regulated, and an average of 5848 lncRNAs (range 3660 to 7510) were down-regulated in HCC tissue (≥ 2.0-fold) (Figure 1E). 1059 lncRNAs were consistently up-regulated, and 1237 were consistently down-regulated in the three pairs of tissue. Among these 2296 consistently dysregulated lncRNAs, NONHSAT071078 (fold change: 12.047939) was one of the most down-regulated lncRNAs (Table 1).

**Table 1:** Up- or down- regulated lncRNAs identified in microarray

Down-regulated lncRNAs		Up-regulated lncRNAs	
lncRNAs	Fold change(T/N)	lncRNAs	Fold change(T/N)
NONHSAT071078	12.047939	NONHSAG015008	15.26645
NONHSAT044093	11.64624	NONHSAT041516	14.26585
NONHSAT102341	10.1019	NONHSAT091472	13.05869
NONHSAT101864	9.343608	NONHSAT028102	11.21455
NONHSAG046953	8.616863	NONHSAT083846	10.00606
NONHSAT030843	7.335756	NONHSAT129787	9.416011
NONHSAG017018	7.078176	NONHSAT005451	7.959627
NONHSAG014397	6.172058	NONHSAT015144	7.074709
NONHSAT093925	5.376635	NONHSAT093866	6.723495
NONHSAT123001	5.012769	NONHSAT041813	5.128702

Three pairs of HCC and their adjacent noncancerous tissue were analyzed by using a custom human lncRNA microarray. Among the lncRNAs consistently up- or down- regulated in all three pairs of samples, the top 10 up- or down-regulated lncRNAs were shown in the table. lncRNA-NONHSAT071078 was named as LncRNA-SVUGP2 in the manuscript.

Next, hierarchical clustering was performed to analyze the patterns of lncRNAs and coding genes in HCC and noncancerous tissue (Figure 1F). Distinguishable lncRNA and mRNA profiling between HCC and noncancerous tissue were observed.

Additionally, GO analysis and KEGG pathway analysis were performed to evaluate the functional categories and involved pathways of the dysregulated coding genes in HCC. The top 12 GO terms, including Cellular Component, Molecular Function, and Biological Process, and pathways are presented (Supplementary Figure 1).

### lncRNA-SVUGP2 is down-regulated in human HCC tissues

**Figure 2:**
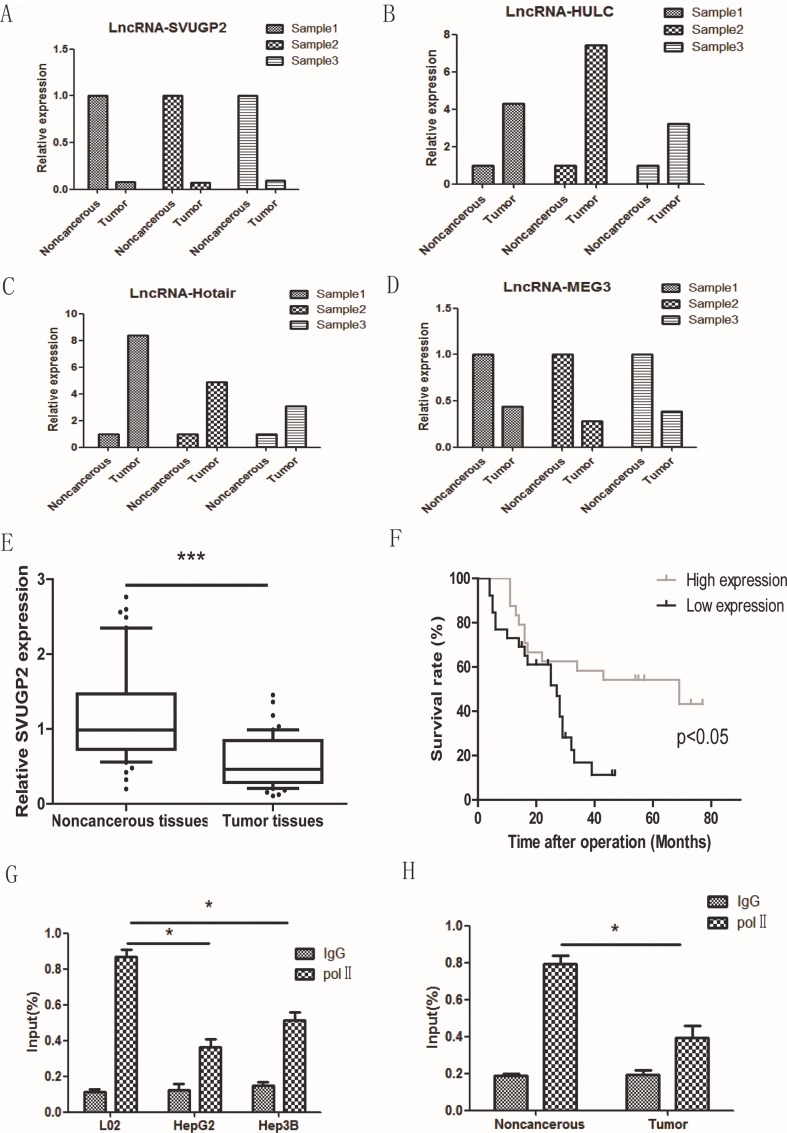
The level of lncRNA-SVUGP2 was decreased in HCC RT-qPCR was performed to test the level of lncRNA-SVUGP2 (**A**) and some reported dysregulated lncRNAs (**B**–**D**) in three HCC samples. (**E**) lncRNA-SVUGP2 level was decreased in HCC tumor tissues compared with that in adjacent noncancerous tissues in 50 additional pairs of tissue samples. (**F**) Kaplan-Meier survival curve of 50 patients with high or low lncRNA-SVUGP2 level. Higher lncRNA-SVUGP2 level is correlated with better prognosis. (**G**) The result of ChIP-qPCR showing RNA Pol II occupancy on lncRNA-SVUGP2 promoter was decreased in HepG2 and Hep3B cell lines compared with that in normal hepatocyte, L02. (**H**) RNA Pol II occupancy on lncRNA-SVUGP2 promoter was also reduced in HCC tumor tissues compared to that in adjacent noncancerous tissues.

lncRNA NONHSAT071078, one of the most down-regulated lncRNA in the microarray, is consistently and significantly decreased in all three pairs of tissues, which attracts our interest to work on this lncRNA (Figure 2A). The reliability of the microarray was further confirmed by checking the levels of some previous reported dysregulated lncRNAs in HCC. Consistent with early studies, lncRNA-HULC and HOTAIR were up-regulated and lncRNA-MEG3 was down-regulated in the HCC samples, suggesting the results of our microarray were reliable (Figure 2B–2D).

LncRNA NONHSAT071078 is located on human chromosome 2 (64068990- 64084752) with a length of 606 bp. This lncRNA was included in NONCODE V4, a publicly comprehensive and systematic ncRNA database [39]. This lncRNA is a splice variant of the UGP2 gene and is thus named lncRNA-SVUGP2. The protein-coding potential of this RNA was further assessed using two different tools: coding potential calculator algorithm and PhyloCSF. Both of these tools suggest that this RNA is likely a non-coding RNA.

To solidify the conclusion that lncRNA-SVUGP2 is down-regulated in HCC, we performed RT-qPCR to measure this lncRNA level in 50 additional pairs of HCC and adjacent noncancerous samples. Consistent with the results of microarray, a significant reduction in lncRNA-SVUGP2 levels was observed in these samples (Figure 2E). Importantly, high level of lncRNA-SVUGP2 was correlated with better survival, indicating it suppresses the progression of HCC (Figure 2F).

The observation that lncRNA-SVUGP2 was down-regulated in HCC may be due to the transcription suppression or other mechanism like splicing defect. To get insight into the molecular mechanism, we tested whether the transcription of lncRNA –SVUGP2 was suppressed in liver cancer cells. For this purpose, we performed a ChIP-qPCR with anti-RNA polymerase II (Pol II) antibody in normal liver cell line L02 and liver cancer cell lines HepG2 and Hep3B and analyzed precipitated DNA by using primers recognizing promoter region of lncRNA-SVUGP2. Compare to normal liver cell line, Pol II occupancy was decreased in two liver cancer cell lines (Figure 2G). The reduction of Pol II occupancy was also observed when compare HCC tissues to noncancerous tissues (Figure 2H).

Furthermore, to investigate the correlation between lncRNA-SVUGP2 level and the cancer status in HCC patients, the clinicopathological features of 50 hepatocellular cancer cases were summarized (Table 2). Among these 50 cases, 9 were female, and 41 were male. In addition, 19 of 50 patients are older than 60 years. Regarding tumor size, 34 patients harbored tumors > 5 cm in diameter. Additionally, clinical TNM stage (tumor, node, metastasis classification) analysis of these HCC patients indicates that 26 cases had stage I or II disease, and 24 cases had stage III disease.

**Table 2:** The relationship between clinicopathological parameters and lncRNA-SVUGP2 expression

Clinicopathological parameters	Case	LncRNA-SVUGP2		*P*
		Low	High	
**Gender**				0.281
** Male**	41	23	18	
** Female**	9	3	6	
**Age**				0.57
** ≤ 60**	31	15	16	
** > 60**	19	11	8	
**Tumor size**				0.069
** ≤ 5**	16	5	11	
** > 5**	34	21	13	
**TNM stage**				0.002
** I–II**	26	8	18	
** III–IV**	24	18	6	
**HBV infection**				0.382
** No**	11	7	4	
** Yes**	39	19	20	
**Cirrhosis**				0.039
** No**	14	4	10	
** Yes**	36	22	14	

The level of LncRNA-SVUGP2 was measured in 50 cases of HCC samples by RT-qPCR, and further normalized to GAPDH. These 50 cases were divided into two groups based on the low (26 cases) or high (24 cases) level of lncRNA-SVUGP2 in HCC samples. X^2^ test and Fisher's exact test test was then used to analyze whether the level of LncRNA-SVUGP2 was significantly changed among patients with different gender, age, tumor size, TNM stage, HBV infection and cirrhosis status.

We divided the patients into two groups according to low or high levels of lncRNA-SVUGP2 in HCC. Statistical analysis shows the level of LncRNA is not significantly changed among patients with different gender, age or tumor size. However, reduced lncRNA levels are noted in patients with higher TNM stages (III or IV), suggesting the role of lncRNA-SVUGP2 is more important in later TNM stages. One explanation is that lncRNA-SVUGP2 may repress various biological processes required for late stage tumor development, such as angiogenesis and metastasis.

### lncRNA-SVUGP2 inhibits proliferation and invasion of liver cancer cells

**Figure 3:**
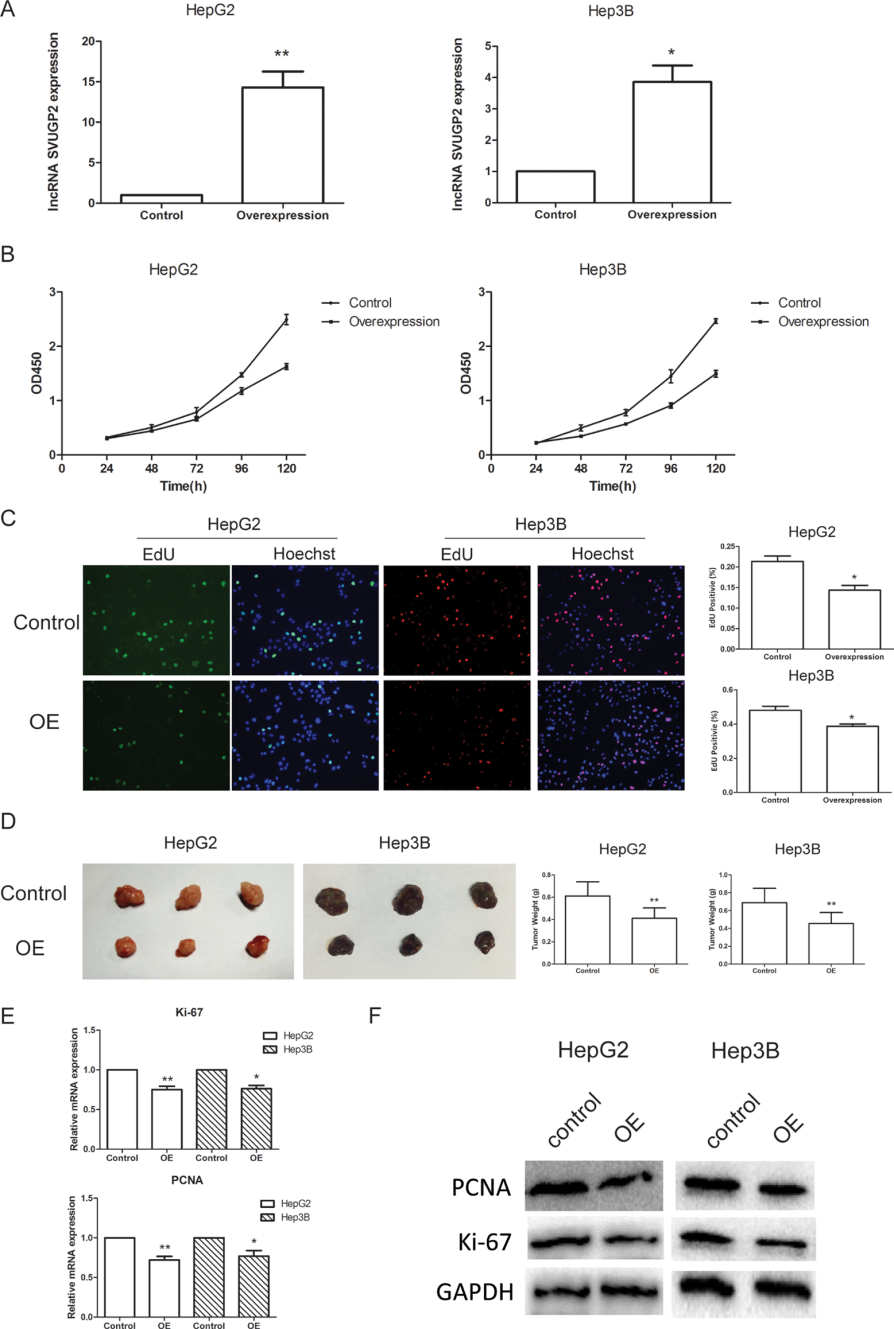
lncRNA-SVUGP2 inhibits proliferation of HepG2 and Hep3B human liver cancer cell lines (**A**) RT-qPCR was used to measure the level of lncRNA-SVUGP2 in control or lncRNA overexpression (OE) group in both HepG2 and Hep3B liver cancer cell line. For control group, cells were infected with lentivirus produced by PGMLV-PA6 empty vector. Cells of OE group were infected with lentivirus encoding lncRNA-SVUGP2. (**B**) Cell proliferation rate of control and OE group was detected by cck-8 assay in both HepG2 and Hep3B cells. (**C**) EdU cell proliferation assay showing overexpression of lncRNA-SVUGP2 suppress cell proliferation. In this assay, EdU is incorporated into cellular DNA, which further reacts with fluorescent reagents. (**D**) To detect cell proliferation rates *in vivo*, we established a xenograft model in nude mice. Control or lncRNA-overexpressing HepG2 or Hep3B cells were injected subcutaneously into nude mice. Five weeks after injection, mice were sacrificed, and tumors were photographed and weighed. qPCR (**E**) and western blot (**F**) was used to detect the mRNA and protein level of Ki-67 and PCNA, two growth markers, in control and lncRNA-overexpressing cells. ^*^p < 0.05, ^**^p < 0.01, relative to control group.

To understand the functional relevance of lncRNA-SVUGP2 to liver cancer carcinogenesis, we tested whether lncRNA regulates cancer cell proliferation and invasion, two processes important for carcinogenesis. HepG2 and Hep3B cell lines stably overexpressing lncRNA-SVUGP2 were generated via lentivirus infection. The overexpression efficiency is confirmed by qPCR (Figure 3A).

The cell proliferation rate was first assessed using the cck-8 assay in these two cell lines (Figure 3B). In both cell lines, lncRNA-SVUGP2 suppresses cell proliferation. This observation was further confirmed by using EdU cell proliferation assay. In this assay, EdU is readily incorporated into cellular DNA during DNA replication, and the labeled DNA was further assessed via its reaction with fluorescent reagents. Consistent with the cck-8 assay, lncRNA-overexpressing cells exhibit reduced EdU staining, suggesting the cell proliferation rate was reduced in these cells (Figure 3C).

The observations of these *in vitro* assays were further confirmed by *in vivo* xenograft model. HepG2 or Hep3B cells with or without lncRNA-SVUGP2 overexpression were injected subcutaneously into nude mice respectively. Five weeks after injection, mice were sacrificed, and tumors were analyzed. Compared to control group, tumors from the lncRNA overexpression group grown more slowly, with smaller sizes and lighter weights observed (Figure 3D), indicating this lncRNA inhibits tumor growth *in vivo*.

Additionally, we compared the mRNA and protein levels of Ki-67 and PCNA, two growth markers, between control cells and cells with lncRNA overexpression. Ki-67 is a nuclear antigen present only in proliferating cells. Proliferating cell nuclear antigen (PCNA) is a nuclear protein that is expressed during cell replication and DNA repair [40]. Both of these genes are highly expressed in HCC, resulting in a high risk of tumor recurrence, more aggressive growth and poor survival [41]. Consistent with the results of proliferation assay, both mRNA and protein levels of these two genes were reduced in lncRNA overexpression group (Figure 3E, 3F).

**Figure 4:**
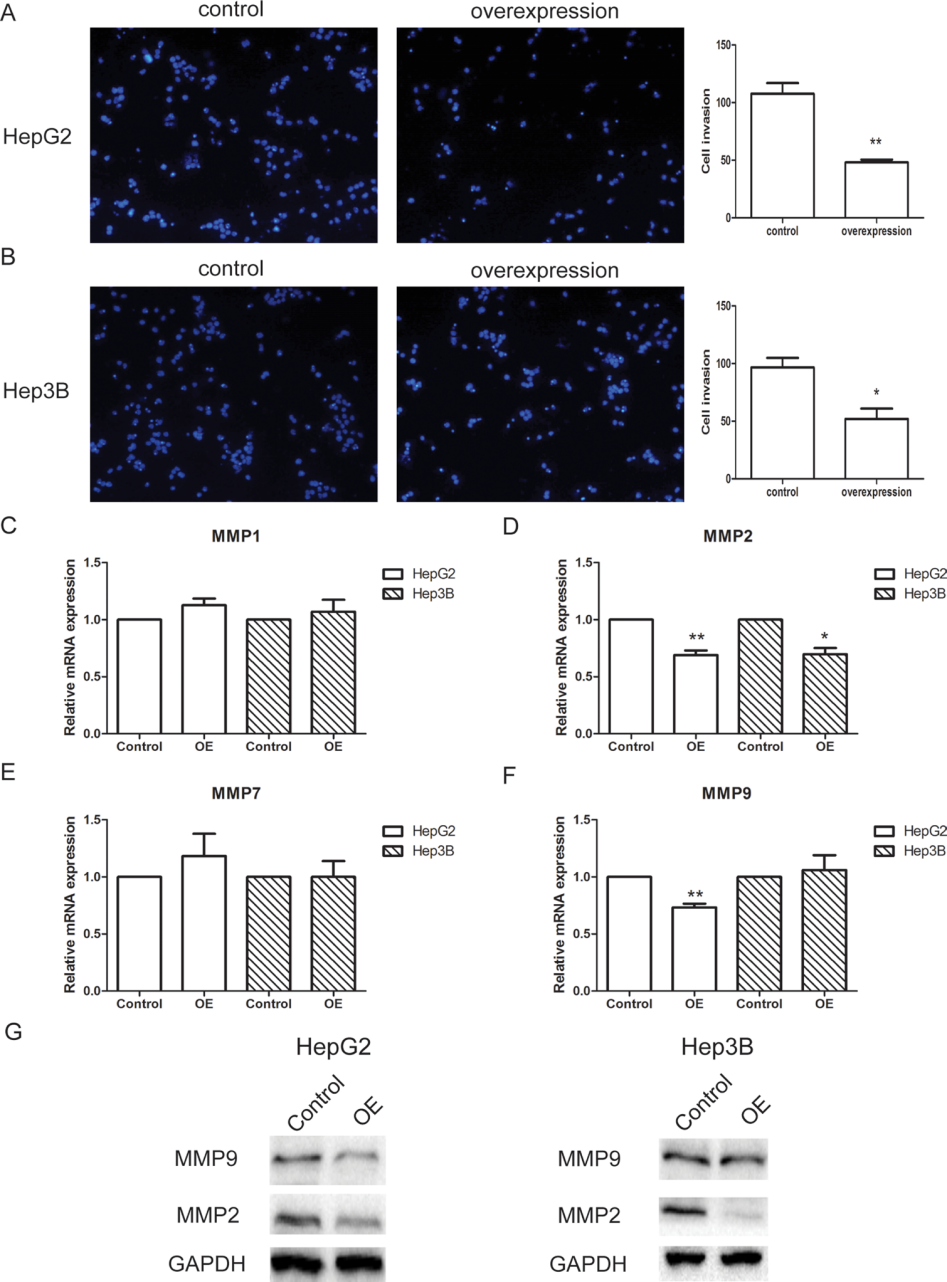
lncRNA-SVUGP2 suppresses invasion ability of HepG2 and Hep3B The invasion ability of control or lncRNA-SVUGP2-overexpressing HepG2 (**A**) or Hep3B (**B**) cells was analyzed by transwell assay. To mimic the tissue barrier *in vivo*, transwell chambers were pre-coated with matrigel. The invasion ability was measured based on the number of the cells that passed through the membrane of the chamber. (**C**–**F**) qPCR was performed to detect the mRNA levels of 4 MMPs (matrix metalloproteinases) in control and lncRNA-overexpressing (OE) cells. This experiment was repeated thrice. ^*^p < 0.05, ^**^p < 0.01. (**G**) Western blot was used to detect MMP-2 and MMP-9 protein levels in HepG2 and Hep3B cells.

The invasion ability of HepG2 and Hep3B cells were measured by transwell assay, with chambers pre-coated with matrigel to mimic tissue barriers *in vivo*. For both cell line, control or lncRNA-SVUGP2-overexpressing cells were added into the upper chamber of the transwell, and the invasion ability was decided by calculating the number of cells passing through the membrane. Overexpression of lncRNA-SVUGP2 inhibits the invasion of these two cell lines (Figure 4A, 4B). Consistent with this observation, mRNA and protein levels of matrix metalloproteinases (MMPs) 2 and 9, enzymes that break down extracellular matrix during metastasis and help cancer cells pass through tissue barriers [42], was reduced in cells with lncRNA overexpression (Figure 4C–4G). In conclusion, overexpression of lncRNA-SVUGP2 results in the suppression of proliferation and invasion in HepG2 and Hep3B liver cancer cells.

### Functional prediction of lncRNA-SVUGP2

**Figure 5:**
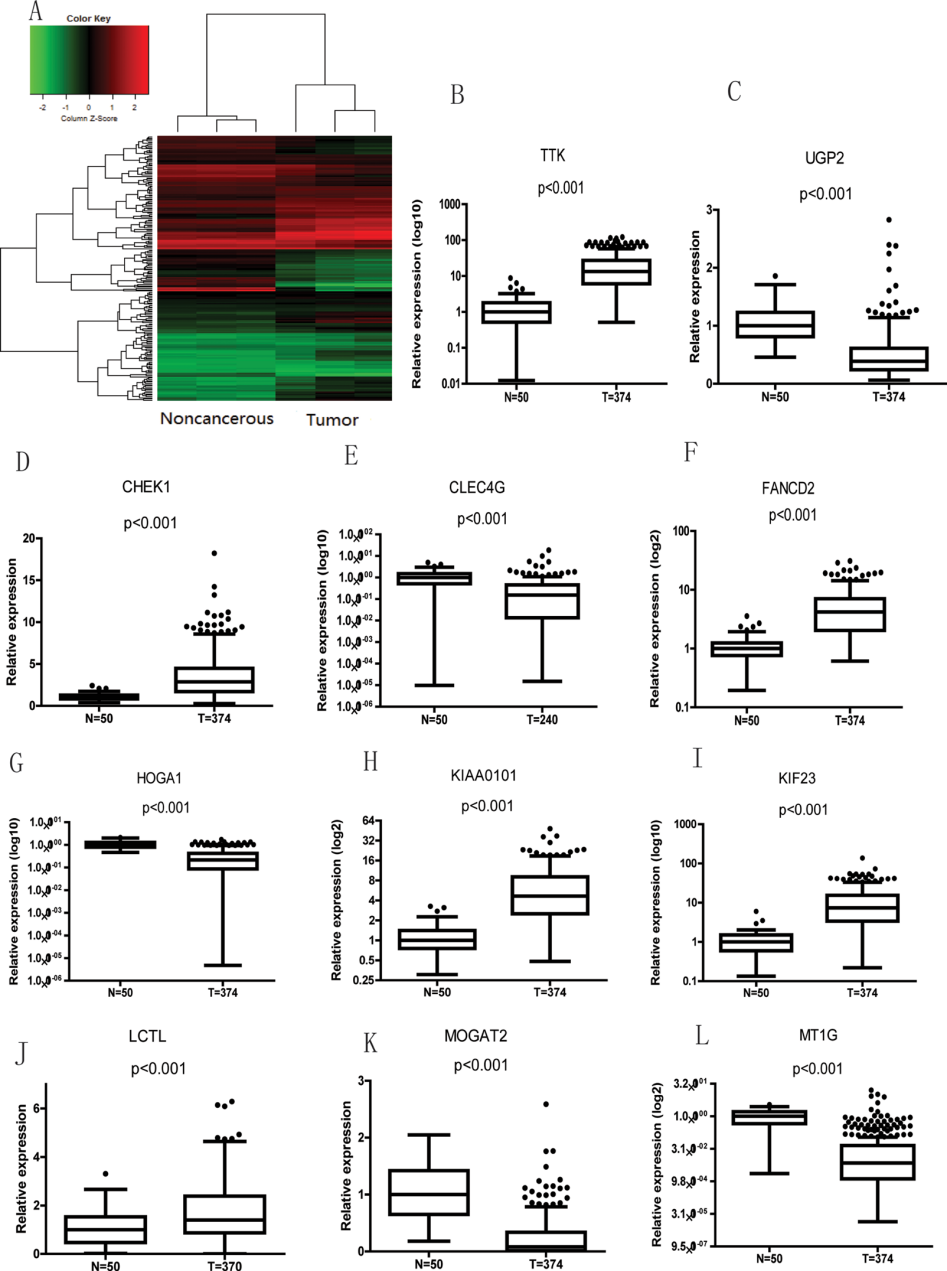
mRNAs correlate with lncRNA-SVUGP2 in noncancerous and liver cancer tissues (**A**) We calculated the correlation of the lncRNA-SVUGP2 expression value with each mRNAs expression value in 3 pairs of noncancerous and HCC tissues. Hierarchically clustered heat map presents the correlation between lncRNA and the top 200 correlated mRNAs. (**B**–**L**) Boxplots showing mRNA levels between noncancerous and cancer tissue in patient sample data from TCGA. The bottom, middle, and top of each box indicate the first, second and third quartiles, respectively. The lower and upper whiskers on the plot represent the 1.5 IQR (inter-quartile range) of the first and third quartiles, respectively.

The results above indicate that lncRNA-SVUGP2 suppresses cell proliferation and invasion in HCC. To better understand the molecular mechanism of lncRNA-SVUGP2 and determine other potential biological processes regulated by this lncRNA, we tried to figure out which mRNAs were regulated by this lncRNA. Briefly, we first calculated the correlation of lncRNA-SVUGP2 level with mRNA levels in 3 pairs of HCC tissues and noncancerous tissues. The mRNAs with correlation *P*-value < 0.05 were regarded as co-expressed mRNAs. A hierarchically clustered heat map of the relationship between samples of the top 200 mRNAs is presented (Figure 5A).

To evaluate the functional categories and pathways involved in the co-expressed mRNAs, we selected the top 200 mRNAs from the list and performed GO analysis and KEGG pathway analysis for the coding genes corresponding to these mRNAs (Supplementary Figure 2). For the GO consortium, the genes were classified among three aspects: cellular component, molecular function, and biological process.

Next, to further narrow down the candidate mRNAs, we selected the most significantly changed mRNAs involved in HCC carcinogenesis among these 200 mRNAs, which is done by choosing the mRNAs that exhibit a > 5-fold change and *P*-value < 0.01 between noncancerous and liver cancer tissues in the microarray (Figure 2D). Using this strategy, 13 mRNAs were selected (Table 3).

**Table 3:** mRNAs correlated with lncRNA-SVUGP2 level and dys-regulated in HCC

GeneSymbol	Regulation	Fold change	*p* value
CLEC4G	down	39.53185	0.002215297
MT1G	down	340.1278	0.006273059
FANCD2	up	7.532945	0.007258298
HIST2H3A	up	21.28292	0.000807
MOGAT2	down	23.25504	0.008000504
LCTL	up	7.262519	0.003426997
CHEK1	up	7.99765	0.0000196
UGP2	down	6.343705	0.009276051
KIAA0101	up	18.94839	0.003486132
KIF23	up	24.93162	0.009041968
TTK	up	19.50945	0.008603159
CENPM	up	18.62225	0.007035683
HOGA1	down	5.456985	0.007846125

The results of mRNA and lncRNA microarray done in 3 pairs of HCC and their adjacent noncancerous tissue were analyzed by Spearman's correlation test. mRNAs closely correlated with lncRNA-SVUGP2 were first selected (*p* < 0.05). Among these candidate mRNAs, 13 mRNAs were dramatically (Fold change > 5) and significantly (*P*-value < 0.01) dysregulated in HCC.

Finally, the clinical correlation of these mRNAs with HCC was tested by using high-throughput data from 50 noncancerous liver tissue and 374 primary HCC tumor samples from The Cancer Genome Atlas (TCGA). 11 of the 13 candidate genes in the last step exhibit statistically significant difference of mRNA levels between patient noncancerous and cancer tissues (Figure 5B–5I). These 11 genes include genes are involved in cell cycle, such as CHEK1, KIF23, and TTK; DNA damage, such as FANCD2 and KIAA0101; and metabolism: HOGA1, LCTL, MOGAT2, MT1G, and UGP2, biological processes important for carcinogenesis.

**Figure 6:**
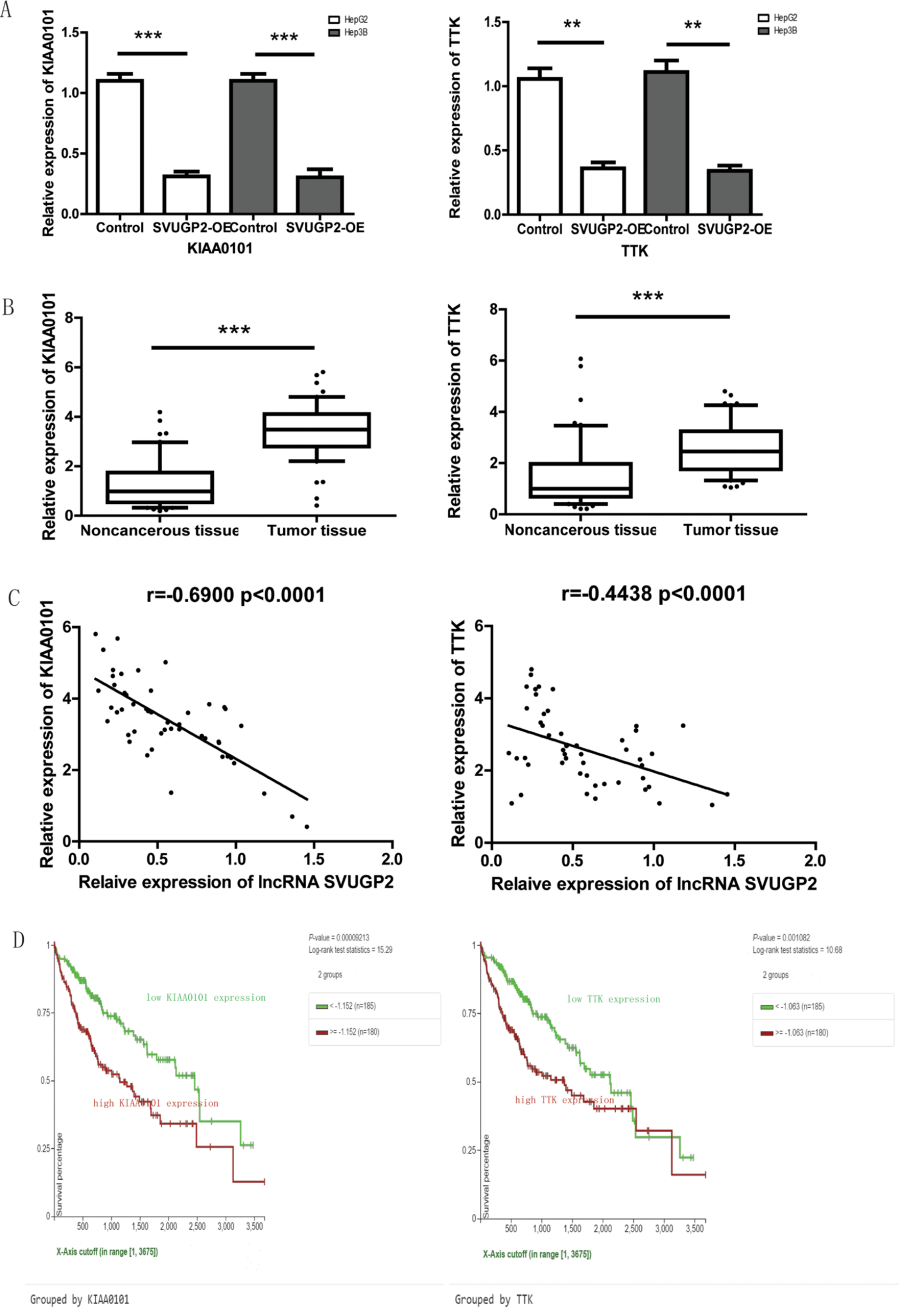
lncRNA-SVUGP2 down-regulates KIAA0101 and TTK in HCC (**A**) RT-qPCR was performed to analyze the mRNA level of KIAA0101 and TTK in HepG2 or Hep3B cells with or without lncRNA-SVUGP2 overexpression. The mRNA level of these two genes was further normalized to GAPDH. (**B**) KIAA0101 and TTK mRNA levels were assessed by qPCR in 50 pairs of liver noncancerous and cancer tissues. ^***^p < 0.001 (**C**) Spearman Rank correlation analysis of the relationship between the lncRNA-SVUGP2 and KIAA0101 or TTK mRNA level. (**D**) Kaplan-Meier survival curves for HCC patients with higher or lower KIAA0101 and TTK mRNA level.

Two genes, KIAA0101 and TTK, were selected among these 11 candidates since both of them have been reported highly expressed in HCC specimens, and regulated carcinogenesis of HCC. KIAA0101 correlates with enhanced metastatic potential and poor prognosis in HCC [43], and TTK promotes the proliferation and migration of HCC cells [44, 45]. Therefore, these two genes are the most likely lncRNA-SVUGP2 targeting genes that further affect hepatocellular carcinogenesis. Consistent with the results of microarray, in both HepG2 and Hep3B cell lines, overexpression of lncRNA suppresses the mRNA level of KIAA0101 and TTK (Figure 6A). Additionally, the mRNA levels of these two genes were analyzed by qPCR in 50 pairs of HCC and adjacent noncancerous samples, the same samples as which used in Figure 2E. Consistent with the previous studies, both KIAA0101 and TTK were up-regulated in HCC (Figure 6B). Moreover, expression of lncRNA-SVUGP2 was tightly inversely correlated with KIAA0101 and weakly correlated with TTK in these 50 pairs of HCC patient samples (Figure 6C). Finally, by using UCSC Xena, we revealed that high expression of KIAA0101 and TTK in HCC tumors was correlated with reduced overall survival in HCC patients (Figure 6D). In conclusion, the level of lncRNA-SVUGP2 is conversely correlated with KIAA0101 and TTK mRNAs, two genes promoting hepatocellular carcinogenesis.

## DISCUSSION

The available data indicate that a number of lncRNA levels are aberrant in HCC, and these lncRNAs play very important roles in the critical biological process of HCC, such as tumor growth, metastasis, and angiogenesis. LncRNA HULC promotes tumor growth via down-regulating its neighbor gene p18 [46]. LncRNA-hPVT1 and UFC1 increase HCC cell proliferation and cell cycling [47, 48]. In addition, lncRNA H19 suppresses the migration of HCC cells by reducing the expression of markers of the epithelial-to-mesenchymal transition [49]. LncRNA HOTAIR enhances cancer cell invasion and metastasis by up-regulating matrix metallopeptidase 9 and vascular endothelial growth factor [33]. Moreover, lncRNA MVIH activates tumor-inducing angiogenesis by inhibiting the secretion of phosphoglycerate kinase 1 (PGK1).

In the result of our microarray, besides the down-regulation of lncRNA-SVUGP2, the dysregulation of several reported lncRNAs was also observed. For instance, lncRNA-HULC and HOTAIR, two reported upregulated lncRNAs, were significantly upregulated by 4–8 folds in HCC tissue. Another reported down-regulated LncRNA-MEG3 was also decreased by 50–70% in HCC tissue (See Figure 2B–2D). The observation that several lncRNAs are up- or down-regulated in HCC tissue suggests these lncRNAs may regulate HCC progression cooperatively. Further studies are needed to figure out the detail molecular mechanism how do these lncRNAs cooperate to regulate carcinogenesis in HCC.

In our study, we found lncRNA-SVUGP2 was significantly down-regulated in HCC, and high level of lncRNA was correlated with better prognosis of HCC patients. Additionally, overexpression of lncRNA-SVUGP2 inhibits the proliferation rate and invasion ability of HCC cells. Consistent with these observations, mRNA and protein levels of the growth markers, Ki-61 and PCNA, and invasion markers, MMP2 and MMP9, were inhibited by lncRNA-SVUGP2, supporting the conclusion that lncRNA suppresses the progression of HCC. Interestingly, lncRNA-SVUGP2 levels correlate with serval mRNAs, 13 of them are dysregulated in HCC. Further studies showed KIAA0101 and TTK, two reported dysregulated mRNAs in HCC, was inversely correlated with lncRNA-SVUGP2, in both liver cancer cell lines and tumor tissue. All these observations suggest, lncRNA-SVUGP2 plays a role in suppress carcinogenesis of HCC, and more than one gene is affected by this lncRNA, which makes the molecular mechanism be more complicate. The following work need to be done is to uncover which are the most important genes involved in the lncRNA-SVUGP2-mediated carcinogenesis suppression. What's more, given that lncRNAs regulating transcription via changing the occupancy of transcriptional factors and RNA polymerase, DNA methylation and histone modification in promoter region [35], it will also be interesting to investigate the epigenetic mechanism how lncRNA-SVUGP2 regulates the transcription of target genes.

A potential value of our research involves the use of lncRNA-SVUGP2 as a new treatment target of HCC in the future. The concept of lncRNA-targeting therapy involves silencing harmful lncRNA that are highly transcribed in the tumor and overexpressing low transcribed lncRNAs with tumor suppressing function, such as lncRNA-SVUGP2. One obstacle for lncRNA-targeting therapy in HCC is the selection of the proper target lncRNA, which should be important and specific. Therefore, high-throughput data detecting global differences in lncRNA and mRNA levels in noncancerous and cancer tissue of HCC is necessary. Although a couple of lncRNAs have been identified as tumorigenesis regulators in HCC, the identification of a lncRNA as the clinical treatment target in HCC is still challenging and will require considerable effort in the future. These results suggest that lncRNA-SVUGP2 may serve as a novel predictor and treatment target of HCC.

## MATERIALS AND METHODS

### RNA preparation and microarray analysis

HCC samples and their corresponding adjacent noncancerous liver tissues were collected from Shanghai Biochip Co. Ltd. This study was approved by the Ethics Committee of the affiliated Tongji hospital of Tongji university, and all patients signed an informed consent form. All the samples are surgical-resected. Each pair contains tumor tissue and its adjacent noncancerous tissue. 39 cases were associated with HBV infection, 6 with HCV infection, and 5 with neither HBV nor HCV. Human tissues were frozen in liquid nitrogen immediately and stored at −80°C freezer until usage. Total RNA was extracted from three pairs of HCC and their adjacent noncancerous tissues using Trizol reagent. OE Biotech human lncRNA Microarray v4.0 4×180K was used to analyze the global profiling of human lncRNAs and protein-coding transcripts in these samples. The microarray contains 78,243 lncRNAs and 32,776 coding genes. The raw data of the microarray were extracted by Feature Extraction software and further quantile normalized using the GeneSpring software. Heat maps were generated by Treeview-Cluster software based on lncRNA and mRNA levels. Gene Ontology (GO) analysis (http://www.geneontology.org) and KEGG pathways (http://www.genome.jp/kegg/) were used for analyzing the genes altered in HCC tissue or co-expressed with lncRNA-SVUGP2. The microarray analysis was performed by OEbiotech.

### Cell culture

293FT cells and Human liver cancer HepG2, Hep3B cells were purchased from ATCC and were cultured in DMEM/F12 medium (Thermo). The medium was supplemented with 10% fetal bovine serum (FBS, Gibco) and 1% penicillin/ streptomycin. Cells were cultured at 37°C in a humidified incubator containing 5% CO_2_.

### Vector constructs, lentivirus package, and infection

lncRNA-SVUGP2 was amplified from the cDNA of HepG2 cells using PrimerSTAR premix (Takara) and cloned into the PGMLV-PA6 lentivirus vector (Genomeditech, Shanghai) using the following primers: Forward (XhoI) CCGCTCGAGCATTGAAGAAATTTAAGTTCGTGTG GTTTTACC, Reverse (BamHI) CCGGGATCCCTCAG GCCAGCTGATCGTTTG. For lentivirus packaging, 293FT cells were cotransfected with PAX2, VSVG and PGMLV using the FuGene transfection reagent (Roche). The medium was harvested at 48 and 72 hours after transfection, then filtered to remove cell debris and used for infection. 2 × 10^5^ HepG2 and Hep3B cells were seeded into six-well plates 1 day before infection. The medium was then replaced with the virus-containing supernatant supplemented with 8 μg/ml polybrene (Genomeditech, Shanghai). 8 hours later, the medium was replaced with fresh medium. The infected cells were selected using 1 μg/ml puromycin.

### Cell counting kit-8 (cck-8) assay

Control or lncRNA-SVUGP2-overexpressing HepG2 and Hep3B cells were seeded in 96-well plates at a density of 2000 cells per well in 100 μl of complete medium and grown overnight. After a certain time point, 10 μl of the cck-8 solution (Dojindo, Tokyo) was added to each well and incubated at 37°C for 1 hour. The absorbance per well was measured at 450 nm using a Microplate Reader. The cell growth curve is generated according to the absorbance of all time points.

### EdU incorporation assay

This assay was analyzed using a Cell-Light™ EdU Cell Proliferation Detection kit (RiboBio, China) according to according to previous methods [50]. Control or lncRNA-SVUGP2-overexpressing HepG2 and Hep3B stable cell lines were seeded at 1.5 × 10^5^ cells/well in 24-well plates. Then, cells were labeled with 5-ethynyl-2′-deoxyuridine (EdU), which incorporates into actively proliferating cells, and fixed with paraformaldehyde. After DNA staining, cellular immunostaining was observed using an epifluorescence microscope (Leica, Germany). Digital images were acquired and analyzed with ImageJ software.

### Transwell assay

Cell invasion was assessed using a transwell assay. Briefly, 5 × 10^4^ HepG2 and Hep3B cells in 100 μL of 0.1% serum medium were placed in the upper chamber of an insert (pore size, 8 μm, Corning) precoated with Matrigel (1:8 dilution, BD). The lower chamber was filled with 600 μL of 10% fetal bovine serum medium. After 24 hours of incubation, the cells on the upper chamber of the filter were removed with a cotton swab, and the cells on the underside were fixed with 4% paraformaldehyde. The fixed cells were stained with Hoechst 33342, and the cells in five randomly selected fields were counted and photographed using a phase contrast microscope.

### Quantitative real-time PCR

Quantitative real-time PCR was performed to study lncRNA-SVUGP2 levels and mRNA levels of various cancer-related genes using SYBR Premix Ex Taq II on the LightCycler 480 system according to the manufacturer's instructions. The quantification analysis for target gene levels was performed using the relative quantification comparative CT method. Primers sequences for qPCR are presented in Table 4. Target gene levels were further normalized to the mRNA level of GAPDH.

**Table 4:** Primers used for RT-qPCR in this study

Name	Forward (5′–3′)	Reverse (5′–3′)
SVUGP2	GGAAGAGAGACCTGCCCTGTAGC	CTTTTCTCAGTCCTCCCGCTTC
Ki-67	TCCTTTGGTGGGCACCTAAGACCTG	TGATGGTTGAGGTCGTTCCTTGATG
PCNA	CCTGCTGGGATATTAGCTCCA	CAGCGGTAGGTGTCGAAGC
MMP1	GGGAGATCATCGGGACAACTC	GGGCCTGGTTGAAAAGCAT
MMP2	GACAACGCCCCCATACCAG	CACTCGCCCCGTGTGTTAGT
MMP7	GAGATGCTCACTTCGATGAGG	GGATCAGAGGAATGTCCCATAC
MMP9	ACGCAGACATCGTCATCCAGT	GGACCACAACTCGTCATCGTC
GAPDH	GGAGCGAGATCCCTCCAAAAT	GGCTGTTGTCATACTTCTCATGG
KIAA0101	AGCTTTGTTGAACAGGCATTT	GGCAGCAGTACAACAATCTAAGC
TTK	TCAAGGAACCTCTGGTGTCA	GGTTACTCTCTGGAACCTCTGGT
HULC	TCATGATGGAATTGGAGCCTT	CTCTTCCTGGCTTGCAGATTG
HOTAIR	CAGTGGGGAACTCTGACTCG	GTGCCTGGTGCTCTCTTACC
MEG3	CTGCCCATCTACACCTCACG	CTCTCCGCCGTCTGCGCTAGGGGCT

### Western blot

Cells were lysed with RIPA buffer (Thermo). Primary antibodies targeting GAPDH (1:5000, Biogot), Ki-67 (1:3000, Abcam), PCNA (1:2000, Abcam), MMP2 (1:2000, Biogot), and MMP9 (1:1500, Biogot) were employed.

### Chromatin immunoprecipitation assay

Chromatin immunoprecipitation was performed using EZ-Magna ChIP^TM^ A/G Kit (Milipore) according to previous methods [51]. DNA immunoprecipitated from the sonicated cell lysates was quantified by SYBR Green Real-time PCR. RNA polymerase II (abcam) was used in this study. PCR primer sequences is shown as follows: PF: 5′-tatgtacgggaagggtcggt-3′, PR:5′-ctatttcaccgcccccaagt-3′

### Tumor xenografts in nude mice

Cell lines stably overexpressing lncRNA-SVUGP2 were generated by lentivirus infection in two human liver cancer cell lines: HepG2 and Hep3B cells. Briefly, 1 × 10^7^ HepG2 cells or 5 × 10^6^ Hep3B cells with or without lncRNA-SVUGP2 were suspended in 200 μL of PBS, mixed with 20 μL Matrigel and injected subcutaneously into the right flank of 4- to 5-week-old nude mice. Mice were sacrificed 5 weeks after injection. For each group, tumors from 3 mice were weighed and photographed.

### TCGA dataset

Differential expression analysis was performed using edgeR [52]. Xena Browser was used to compare the different Kaplan–Meier curves using the log-rank test (http://xena.ucsc.edu/). The expression values were normalized across cancer types. Red represents high gene expression values, whereas green represents low gene expression.

### Statistical analysis

Statistical analysis was conducted using SPSS 22.0 or Graphpad. The significance of group differences for normally distributed data was assessed with Student's *t* test. Wilcoxon signed rank test was used for Figure 2E, Figure 6B. Kaplan-Meier and Log-Rank test was used for Figure 2F, Figure 6D. Spearman's correlation test was used in Figure 6C. Mann-Whitney *U* test was used in Figure 5D–5L. X^2^ test and Fisher's exact test were used in Table 2. A level of *P* ≤ 0.05 was accepted as statistically significant. Data are presented as means ± SEs.

### Ethical approval and informed consent

This study was conducted in accordance with the amended Declaration of Helsinki. All study methods, including those for both the human and animal studies, were approved by the Institutional Review Board and Ethics Committee of the Shanghai Biochip and Tongji University. All subjects enrolled into the study provided written informed consent to participate.

## SUPPLEMENTARY MATERIALS FIGURES



## References

[1] Venook AP, Papandreou C, Furuse J, de Guevara LL (2010). The incidence and epidemiology of hepatocellular carcinoma: a global and regional perspective. Oncologist.

[2] Jemal A, Bray F, Center MM, Ferlay J, Ward E, Forman D (2011). Global cancer statistics. CA Cancer J Clin.

[3] Chiang JK, Koo M, Kuo TB, Fu CH (2010). Association between cardiovascular autonomic functions and time to death in patients with terminal hepatocellular carcinoma. J Pain Symptom Manage.

[4] Torre LA, Bray F, Siegel RL, Ferlay J, Lortet-Tieulent J, Jemal A (2015). Global cancer statistics, 2012. CA Cancer J Clin.

[5] Kaplowitz N Mechanisms of liver cell injury. J Hepatol. 2000 (Suppl).

[6] Song P, Feng X, Zhang K, Song T, Ma K, Kokudo N, Dong J, Yao L, Tang W (2013). Screening for and surveillance of high-risk patients with HBV-related chronic liver disease: promoting the early detection of hepatocellular carcinoma in China. Biosci Trends.

[7] Aravalli RN, Cressman EN, Steer CJ (2013). Cellular and molecular mechanisms of hepatocellular carcinoma: an update. Arch Toxicol.

[8] Shin JW, Chung YH (2013). Molecular targeted therapy for hepatocellular carcinoma: current and future. World J Gastroenterol.

[9] Cao C, Sun J, Zhang D, Guo X, Xie L, Li X, Wu D, Liu L (2015). The long intergenic noncoding RNA UFC1, a target of MicroRNA 34a, interacts with the mRNA stabilizing protein HuR to increase levels of beta-catenin in HCC cells. Gastroenterology.

[10] Sherwood V (2015). WNT signaling: an emerging mediator of cancer cell metabolism?. Mol Cell Biol.

[11] Xie H, Ma H, Zhou D (2013). Plasma HULC as a promising novel biomarker for the detection of hepatocellular carcinoma. BioMed Res Int.

[12] Yang Z, Zhou L, Wu LM, Lai MC, Xie HY, Zhang F, Zheng SS (2011). Overexpression of long non-coding RNA HOTAIR predicts tumor recurrence in hepatocellular carcinoma patients following liver transplantation. Ann Surg Oncol.

[13] Ishibashi M, Kogo R, Shibata K, Sawada G, Takahashi Y, Kurashige J, Akiyoshi S, Sasaki S, Iwaya T, Sudo T, Sugimachi K, Mimori K, Wakabayashi G, Mori M (2013). Clinical significance of the expression of long non-coding RNA HOTAIR in primary hepatocellular carcinoma. Oncol Rep.

[14] Quagliata L, Matter MS, Piscuoglio S, Arabi L, Ruiz C, Procino A, Kovac M, Moretti F, Makowska Z, Boldanova T, Andersen JB, Hämmerle M, Tornillo L (2014). Long noncoding RNA HOTTIP/HOXA13 expression is associated with disease progression and predicts outcome in hepatocellular carcinoma patients. Hepatology.

[15] Liu YR, Tang RX, Huang WT, Ren FH, He RQ, Yang LH, Luo DZ, Dang YW, Chen G (2015). Long noncoding RNAs in hepatocellular carcinoma: novel insights into their mechanism. World J Hepatol.

[16] He Y, Meng XM, Huang C, Wu BM, Zhang L, Lv XW, Li J (2014). Long noncoding RNAs: novel insights into hepatocelluar carcinoma. Cancer Lett.

[17] Lai MC, Yang Z, Zhou L, Zhu QQ, Xie HY, Zhang F, Wu LM, Chen LM, Zheng SS (2012). Long non-coding RNA MALAT-1 overexpression predicts tumor recurrence of hepatocellular carcinoma after liver transplantation. Med Oncol.

[18] Qiu MT, Hu JW, Yin R, Xu L (2013). Long noncoding RNA: an emerging paradigm of cancer research. Tumour Biol.

[19] Shibata C, Otsuka M, Kishikawa T, Ohno M, Yoshikawa T, Takata A, Koike K (2015). Diagnostic and therapeutic application of noncoding RNAs for hepatocellular carcinoma. World J Hepatol.

[20] Ding C, Yang Z, Lv Z, Du C, Xiao H, Peng C, Cheng S, Xie H, Zhou L, Wu J, Zheng S (2015). Long non-coding RNA PVT1 is associated with tumor progression and predicts recurrence in hepatocellular carcinoma patients. Oncol Lett.

[21] Deng L, Yang SB, Xu FF, Zhang JH (2015). Long noncoding RNA CCAT1 promotes hepatocellular carcinoma progression by functioning as let-7 sponge. J Exp Clin Cancer Res.

[22] Zhu HQ, Zhou X, Chang H, Li HG, Liu FF, Ma CQ, Lu J (2015). Aberrant Expression of CCAT1 Regulated by c-Myc Predicts the Prognosis of Hepatocellular Carcinoma. Asian Pac J Cancer Prev.

[23] Braconi C, Kogure T, Valeri N, Huang N, Nuovo G, Costinean S, Negrini M, Miotto E, Croce CM, Patel T (2011). microRNA-29 can regulate expression of the long non-coding RNA gene MEG3 in hepatocellular cancer. Oncogene.

[24] Li CH, Chen Y (2013). Targeting long non-coding RNAs in cancers: progress and prospects. Int J Biochem Cell Biol.

[25] Wang K, Song Y, Liu W, Wu X, Zhang Y, Li S, Kang L, Tu J, Zhao K, Hua W, Yang C (2017). The noncoding RNA linc-ADAMTS5 cooperates with RREB1 to protect from intervertebral disc degeneration through inhibiting ADAMTS5 expression. Clin Sci (Lond).

[26] Panchapakesan U, Pollock C (2016). Long non-coding RNAs-towards precision medicine in diabetic kidney disease?. Clin Sci (Lond).

[27] Zhang F, Zhang L, Zhang C (2016). Long noncoding RNAs and tumorigenesis: genetic associations, molecular mechanisms, and therapeutic strategies. Tumour Biol.

[28] Cheetham SW, Gruhl F, Mattick JS, Dinger ME (2013). Long noncoding RNAs and the genetics of cancer. Br J Cancer.

[29] Li G, Zhang H, Wan X, Yang X, Zhu C, Wang A, He L, Miao R, Chen S, Zhao H (2014). Long noncoding RNA plays a key role in metastasis and prognosis of hepatocellular carcinoma. BioMed Res Int.

[30] Maass PG, Luft FC, Bähring S (2014). Long non-coding RNA in health and disease. J Mol Med (Berl).

[31] Tripathi V, Shen Z, Chakraborty A, Giri S, Freier SM, Wu X, Zhang Y, Gorospe M, Prasanth SG, Lal A, Prasanth KV (2013). Long noncoding RNA MALAT1 controls cell cycle progression by regulating the expression of oncogenic transcription factor B-MYB. PLoS Genet.

[32] Zhang A, Xu M, Mo YY (2014). Role of the lncRNA-p53 regulatory network in cancer. J Mol Cell Biol.

[33] Geng YJ, Xie SL, Li Q, Ma J, Wang GY (2011). Large intervening non-coding RNA HOTAIR is associated with hepatocellular carcinoma progression. J Int Med Res.

[34] Cheng C, Moore J, Greene C (2014). Applications of bioinformatics to non-coding RNAs in the era of next-generation sequencing. Pac Symp Biocomput.

[35] Kugel JF, Goodrich JA (2013). The regulation of mammalian mRNA transcription by lncRNAs: recent discoveries and current concepts. Epigenomics.

[36] Brockdorff N (2013). Noncoding RNA and Polycomb recruitment. RNA.

[37] Merry CR, Forrest ME, Sabers JN, Beard L, Gao XH, Hatzoglou M, Jackson MW, Wang Z, Markowitz SD, Khalil AM (2015). DNMT1-associated long non-coding RNAs regulate global gene expression and DNA methylation in colon cancer. Hum Mol Genet.

[38] Yoon JH, Abdelmohsen K, Gorospe M (2014). Functional interactions among microRNAs and long noncoding RNAs. Semin Cell Dev Biol.

[39] Xie C, Yuan J, Li H, Li M, Zhao G, Bu D, Zhu W, Wu W, Chen R, Zhao Y (2014). NONCODEv4: exploring the world of long non-coding RNA genes. Nucleic Acids Res.

[40] Ng IO, Chung LP, Tsang SW, Lam CL, Lai EC, Fan ST, Ng M (1994). p53 gene mutation spectrum in hepatocellular carcinomas in Hong Kong Chinese. Oncogene.

[41] Stroescu C, Dragnea A, Ivanov B, Pechianu C, Herlea V, Sgarbura O, Popescu A, Popescu I (2008). Expression of p53, Bcl-2, VEGF, Ki67 and PCNA and prognostic significance in hepatocellular carcinoma. J Gastrointestin Liver Dis.

[42] Verma RP, Hansch C (2007). Matrix metalloproteinases (MMPs): chemical-biological functions and (Q)SARs. Bioorg Med Chem.

[43] Yuan RH, Jeng YM, Pan HW, Hu FC, Lai PL, Lee PH, Hsu HC (2007). Overexpression of KIAA0101 predicts high stage, early tumor recurrence, and poor prognosis of hepatocellular carcinoma. Clin Cancer Res.

[44] Liu X, Liao W, Yuan Q, Ou Y, Huang J (2015). TTK activates Akt and promotes proliferation and migration of hepatocellular carcinoma cells. Oncotarget.

[45] Xu Q, Xu Y, Pan B, Wu L, Ren X, Zhou Y, Mao F, Lin Y, Guan J, Shen S, Zhang X, Wang C, Zhong Y (2016). TTK is a favorable prognostic biomarker for triple-negative breast cancer survival. Oncotarget.

[46] Du Y, Kong G, You X, Zhang S, Zhang T, Gao Y, Ye L, Zhang X (2012). Elevation of highly up-regulated in liver cancer (HULC) by hepatitis B virus X protein promotes hepatoma cell proliferation via down-regulating p18. J Biol Chem.

[47] Wang F, Yuan JH, Wang SB, Yang F, Yuan SX, Ye C, Yang N, Zhou WP, Li WL, Li W, Sun SH (2014). Oncofetal long noncoding RNA PVT1 promotes proliferation and stem cell-like property of hepatocellular carcinoma cells by stabilizing NOP2. Hepatology.

[48] Liu H, Gong M, French BA, Li J, Tillman B, French SW (2014). Mallory-Denk Body (MDB) formation modulates Ufmylation expression epigenetically in alcoholic hepatitis (AH) and non-alcoholic steatohepatitis (NASH). Exp Mol Pathol.

[49] Zhang L, Yang F, Yuan JH, Yuan SX, Zhou WP, Huo XS, Xu D, Bi HS, Wang F, Sun SH (2013). Epigenetic activation of the MiR-200 family contributes to H19-mediated metastasis suppression in hepatocellular carcinoma. Carcinogenesis.

[50] Duan B, Hu J, Zhang T, Luo X, Zhou Y, Liu S, Zhu L, Wu C, Liu W, Chen C, Gao H (2017). miRNA-338-3p/CDK4 signaling pathway suppressed hepatic stellate cell activation and proliferation. BMC Gastroenterol.

[51] Hu J, Chen C, Liu Q, Liu B, Song C, Zhu S, Wu C, Liu S, Yu H, Yao D, Kang J, Zhu L (2015). The role of the miR-31/FIH1 pathway in TGF-β-induced liver fibrosis. Clin Sci (Lond).

[52] Robinson MD, McCarthy DJ, Smyth GK (2010). edgeR: a Bioconductor package for differential expression analysis of digital gene expression data. Bioinformatics.

