# Correction: LncRNA-SVUGP2 suppresses progression of hepatocellular carcinoma

**DOI:** 10.18632/oncotarget.25888

**Published:** 2018-07-27

**Authors:** Jiangfeng Hu, Chenlin Song, Bensong Duan, Xiaoyan Zhang, Dongliang Li, Liang Zhu, Hengjun Gao

**Affiliations:** ^1^ Department of Gastroenterology, Tongji Hospital, Tongji University School of Medicine, Shanghai, China; ^2^ Division of Molecular Biology of the Cell II, German Cancer Research Center, DKFZ-ZMBH Alliance, Heidelberg, Germany; ^3^ Shanghai Engineering Center for Molecular Medicine, National Engineering Center for Biochip at Shanghai, Shanghai, China; ^4^ Department of Hepatobiliary Medicine, Fuzhou General Hospital of Nanjing Command, PLA, Fuzhou, China; ^5^ Department of Gastroenterology, Changzheng Hospital, Second Military Medical University, Shanghai, China; ^6^ National Engineering Center for Biochip at Shanghai, Shanghai, China; ^7^ Department of Gastroenterology, Institute of Digestive Diseases, Tongji University School of Medicine, Shanghai, China

**This article has been corrected:** The correct city name is given below:

^7^ Department of Gastroenterology, Institute of Digestive Diseases, Tongji University School of Medicine, Shanghai, China

Original article: Oncotarget. 2017; 8:97835-97850. https://doi.org/10.18632/oncotarget.18279

