# Supplementary material for: LncRNA-SVUGP2 suppresses progression of hepatocellular carcinoma
Source: Oncotarget. 2017 May 29;8(58):97835–50. doi: 10.18632/oncotarget.18279 (PMC5716695; doi:10.18632/oncotarget.18279)
Supplement: Supplementary file 1 [file oncotarget-08-97835-s001.pdf]

# LncRNA-SVUGP2 suppresses progression of hepatocellular carcinoma

## Supplementary Materials

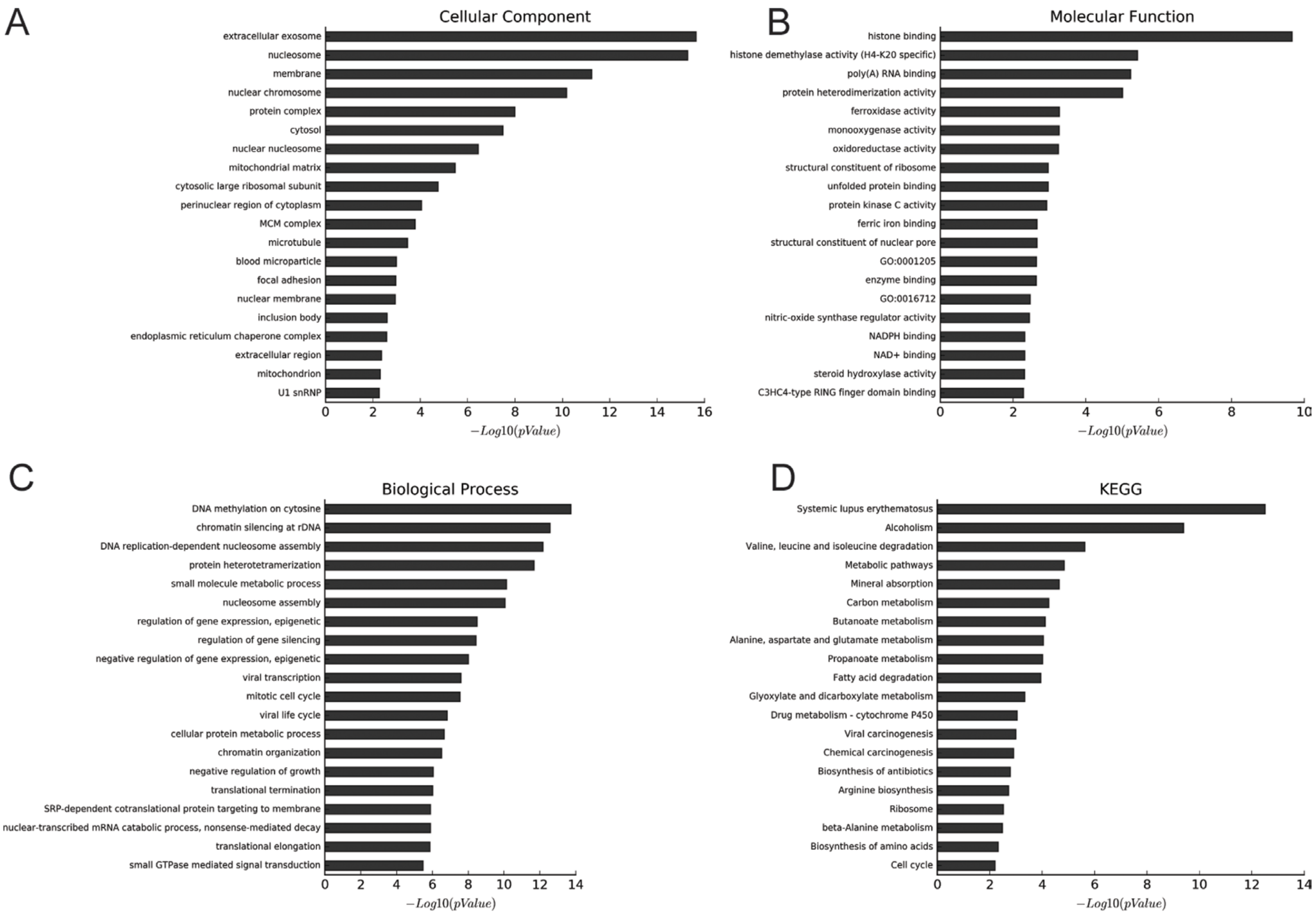


**Supplementary Figure 1: GO analysis and KEGG pathway analysis were performed to evaluate the functional categories and involved pathways of the dysregulated coding genes in HCC.**

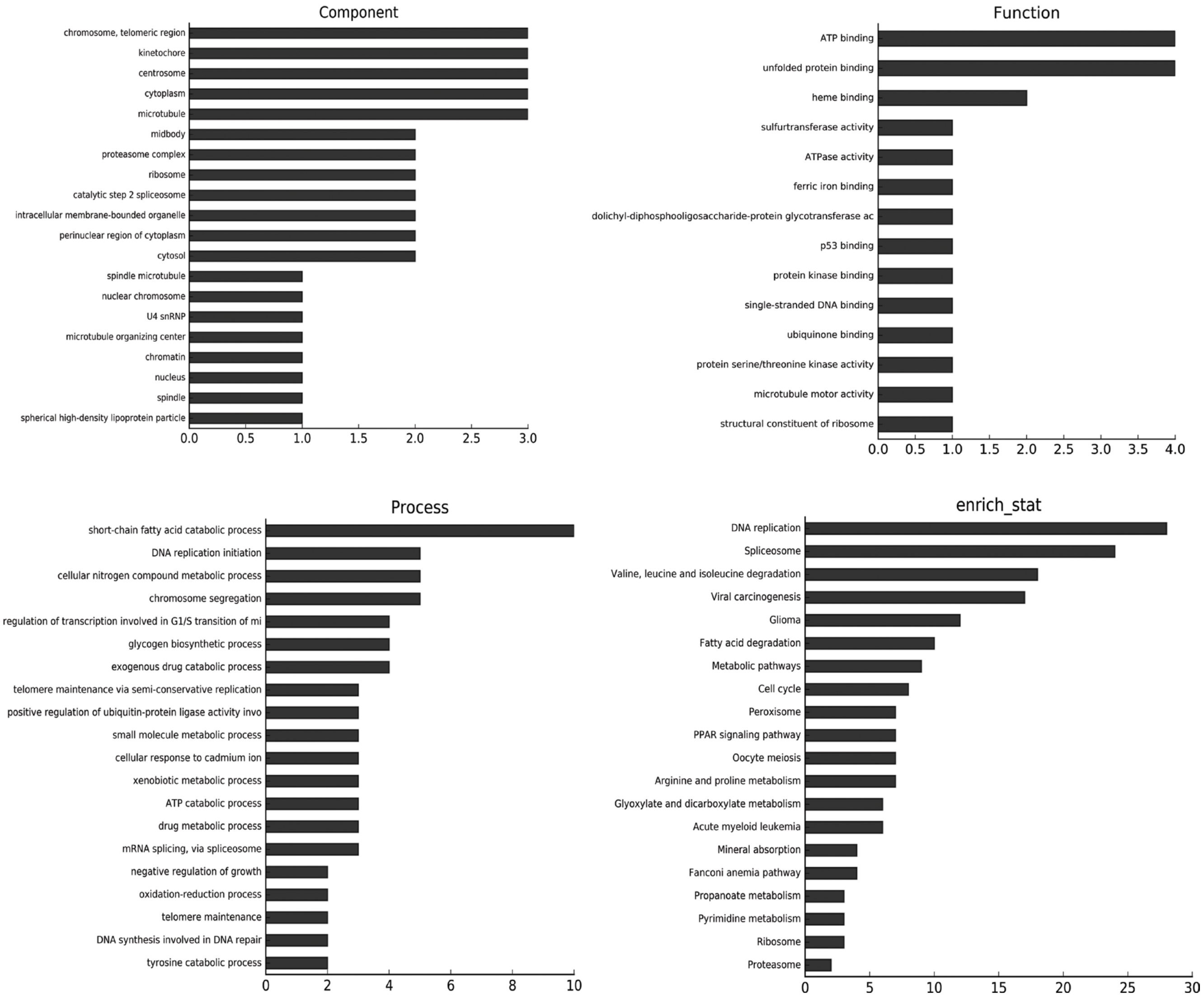


**Supplementary Figure 2: GO analysis and KEGG pathway analysis for the top 200 co-expressed mRNAs of the lncRNA-SVUGP2.**
